# Effects of Sodium Alginate on the Physical Properties and Storage Stability of Freeze-Dried Tofu Coagulated with Crab Shell Extract

**DOI:** 10.3390/foods13010074

**Published:** 2023-12-25

**Authors:** Ga-Yang Lee, Min-Jeong Jung, Byoung-Mok Kim, Joon-Young Jun

**Affiliations:** Food Convergence Research Division, Korea Food Research Institute, Wanju 55365, Republic of Korea; rkdid0925@gmail.com (G.-Y.L.); ooojmj@nate.com (M.-J.J.); bmkim@kfri.re.kr (B.-M.K.)

**Keywords:** freeze-dried tofu, crab shell waste, coagulant, sodium alginate, textural properties, storage stability

## Abstract

The amount of processed by-products such as crab shells is increasing, but industrial utilization is insufficient. In our previous study, crab shell extract (CSE) acted as a coagulant for tofu manufacturing. This study aimed to reduce freeze-dried (FD) tofu breakdown by improving its physical properties through adding sodium alginate (SA). FD state in tofu helps increase storage and availability, but FD tofu frequently fractures during processing, which is a concern for manufacturers. Tofu samples were prepared with either crab shell extract (CSE) or MgCl_2_, and SA, and freeze-dried. In the yields of FD tofu samples, there were no significant differences (*p* < 0.05). The brokenness of FD tofu samples was lower in CSE than in MgCl_2_ and was significantly reduced by SA in both tofu samples, which was affected by hardness. The water-holding capacity decreased after freeze-drying, and CSE reduced this decrease, regardless of SA addition. The microstructures differed depending on the coagulant and were dense upon SA addition. The FD tofu was packed into a multilayer film and stored at 25 °C or 45 °C for 6 months to investigate storage stability. During the storage, brokenness was unchanged in all tofu samples, indicating that they maintained their original structure. There were no significant differences in the volatile base nitrogen and thiobarbituric acid values according to the coagulant type and SA addition (*p* < 0.05). In conclusion, SA reduced FD tofu breakdown by improving the network structure, which may help increase FD tofu quality and decrease economic loss.

## 1. Introduction

The preference for vegetable-derived protein products is increasing owing to the growing vegan market and the imbalance between global population growth and limited resources [1]. Among vegetable proteins, soy protein is rich in essential amino acids, such as lysine, valine, leucine, and methionine, and non-essential amino acids, such as arginine, cysteine, and glutamine [2]. Soy protein is mainly consumed as tofu, accounting for approximately 90% of Asia’s total soy protein consumption [3]. Tofu contains high-quality proteins, lipids, vitamins, minerals, and isoflavones and provides a balanced diet that potentially reduces the risk of cardiovascular disease, diabetes, and hypertension [4].

In tofu production, protein content, temperature, pH, and coagulant type are critical factors that affect textural properties [4]. The main components of soybean protein are classified into 2S, 7S (β-conglycinin), 11S (glycinin), and 15S globulins according to the precipitation coefficient, of which 7S and 11S globulins form protein filaments via thermal denaturation, and it loses the hydrophilic and negatively charged nature of the surface [5,6,7]. The negative charge of the protein molecules is neutralized by divalent cations in coagulants, and the neutralized protein molecules aggregate to form a protein network [8].

Tofu may provide good conditions for microbial growth as it has high moisture and protein contents [9]. Tofu is generally distributed for only a few days under refrigerated conditions, making it difficult to distribute and sell [4]. Recently, the convenience food market has grown in the Republic of Korea owing to the increase in double-income families and single-person households and the consequences of the COVID-19 pandemic [10]. Freeze-drying enables long-term storage and maintains the quality of appearance, nutrients, and flavor, making it possible to produce convenient products [11]. However, the recovery rate through rehydration and cracks produced by the internal expansion of water during freezing affect product quality in the production of freeze-dried (FD) products [5]. The breakdown of FD tofu frequently occurs during processing, which affects product quality and consequently results in economic losses. This breakdown was considered to be caused by the structure and gel strength of tofu. 

Alginate is an anionic polysaccharide obtained from brown algae and is composed of β-D-mannuronic acid (M) and α-L-guluronic acid (G) linked by a bond (1→4) [6]. In particular, alginate has rich functional groups such as hydroxyl and carboxyl groups that can be easily modified to other functional groups and enhance its physical properties by combining with other biopolymers, such as proteins [12]. Alginate can form gels with divalent or multivalent cations; however, the effects of pH, temperature, and hydrophobic interactions on gelation are relatively weak. Notably, Ca^2+^ was extensively studied and examined for its gelation mechanisms, gel properties, influencing factors, and applications in the food industry [13].

Annual crustacean production has consistently increased over the past 20 years according to the Global Production Statistics of the Food and Agriculture Organization of the United Nations [14]. With consumption, 600–800 million tons of crab shells are annually dumped as biowaste worldwide [15]. Crab shells generally contain 30–40% protein, 30–50% calcium carbonate, and 20–30% chitin, with species, part, and seasonal variations [10,16]. Several studies demonstrate the possibility of utilizing crab shell as a calcium-rich value-added material in medical, electronic, and polymer composite preparations [15]. Our previous study focused on the high mineral content in crab shells and the preparation of an acetic acid extract from the shell of a red snow crab (*Chionoecetes japonicus*) to recover the high calcium content. Crab shell extract (CSE) is an excellent coagulant that is comparable to commercial coagulants MgCl_2_ and glucono-δ-lactone in tofu yield and sensorial acceptability [17].

This study aimed to reduce the breakdown of FD tofu with CSE by improving its physical properties through the addition of sodium alginate (SA). To investigate the effects of sodium alginate on the physical properties, hardness, brokenness, and microstructure of FD tofu with CSE, its water-holding capacity and textural properties after rehydration were compared with those of FD tofu with MgCl_2_ (commercial coagulant). In addition, the storage stabilities at 25 °C or 45 °C of the FD tofu samples were evaluated by analyzing the physicochemical parameters. 

## 2. Materials and Methods

### 2.1. Materials

Soybeans (*Glycine max*) were purchased from a local market. Crab shells (carapace, *Chionoecetes japonicus*) were obtained from Yangyang Fisheries (Yangyang, Republic of Korea) and extracted with 3% acetic acid, according to a previous method described by [17]. Crab shell extract (CSE) was used as a coagulant for tofu preparation. Commercial coagulant MgCl_2_ was purchased from Ronic Co. (Paju, Republic of Korea), and food-grade sodium alginate (SA) was purchased from ES Food Ingredient Co., Ltd. (Gunpo, Republic of Korea).

### 2.2. Tofu Preparation

Tofu was prepared by the method of [18], with slight modifications. Soybeans (125 g) were soaked in tap water for 36 h, drained, and ground in 750 mL of tap water using colloid meal. The mash was filtered using a cotton cloth and pressed to obtain soymilk. After adjusting the Brix to 10° of the soymilk with tap water, it was heated to 90 °C for 15 min with stirring. Meanwhile, 2% MgCl_2_ or CSE (*w*/*w* soybean) was added as a coagulant, with or without 0.3% SA (*w*/*w* soybean). The coagulum was cooled, filtered using a two-layered gauze to separate the soybean curd, and freeze-dried. The total weights of tofu and freeze-dried tofu (FD tofu) were determined to calculate yields.

### 2.3. Moisture Content and pH Values

The tofu moisture content was determined using AOAC [19]. The pH of 10° Brix soymilk was measured using a pH meter (SevenEasy S20K; Mettler Toledo International Inc., Columbus, OH, USA).

### 2.4. Brokenness and Hardness

A 20 g sample was placed in a plastic bag and shaken at 50 rpm for 1 min using a stomacher (A2-ST; BNFKOREA Co., Ltd., Gimpo, Republic of Korea) to measure the brokenness of FD tofu. The shaken sample was sieved using a 2500 µm pore size to separate the broken parts of the sample, which were weighed for calculation of brokenness (%, *w*/*w*). The hardness of FD tofu was measured using a texture analyzer (TAXT plus, Stable Micro Systems Ltd., Godalming, UK) equipped with a cylindrical probe (P/5, 5 mm in diameter). The FD tofu was gently cut into cubes (1 × 1 × 1 cm) and compressed up to 25% at a test speed of 2.0 mm/s.

### 2.5. Water-Holding Capacity (WHC)

For determination of water-holding capacity, a 20 g tofu sample was collected. In the case of FD tofu, it was rehydrated with 80 °C tap water for 5 min, cooled, and 20 g of the rehydrated tofu was also collected. The water release of tofu or the rehydrated tofu was measured using a centrifuge filter tube (CTF-CA045-03; Chrom Tech Inc., Apple Valley, MN, USA) [20]. The sample was centrifuged at 4000× *g* for 10 min, the released water was weighed, and the WHC was calculated as the percentage of the released water weight per the original sample weight.

### 2.6. Measuring Texture after Rehydration

The FD tofu was rehydrated at 80 °C for 5 min and drained at room temperature for 30 min. The textural properties of tofu and FD tofu were characterized using a texture analyzer (TAXT Plus, Stable Micro Systems Ltd.), as described by [21], with slight modifications. The tofu and FD tofu were cut into cubes (1.5 × 1.5 × 1.5 cm) and compressed twice up to 30% of the original tofu thickness with a radiused cylinder probe (P/0.5, 12.7 mm in diameter). The test speed was adjusted to 2.0 mm/s, and the hardness, springiness, cohesiveness, and gumminess were measured.

### 2.7. Scanning Electron Microscopy

The FD tofu microstructure was observed using a scanning electron microscope (SEM; Nanoeye, SNE-3000M; SEC Co., Ltd., Suwon, Republic of Korea). A lump of FD tofu was platinum-coated and observed at ×300 magnification and 30 kV.

### 2.8. Storage Stability

Changes in brokenness, volatile base nitrogen (VBN), thiobarbituric acid (TBA) levels, and surface color were determined during FD tofu storage. Each 25 g FD tofu was packed into a multilayer film (polyethylene terephthalate/aluminum/polyethylene/low-density polyethylene, 80 µm thickness) and stored at 25 °C or 45 °C for 6 months. Freeze-dried tofu was collected at 0, 2, 4, 8, 16, and 24 weeks and analyzed.

### 2.9. Volatile Base Nitrogen Value

The VBN value was determined using a Conway unit according to a previous method by [22] with slight modifications. One gram of crushed FD tofu was homogenized (10,000 rpm for 2 min) in 17 mL of deionized water and 2 mL of 20% trichloroacetic acid was added. After homogenization (10,000 rpm for 2 min), the samples were filtered through No. 5C filter paper (Advantec Toyo Kaisha, Ltd., Tokyo, Japan). An aliquot was used to determine the VBN value, which was expressed as milligram percent.

### 2.10. Thiobarbituric Acid Value

The TBA value was determined using the distillation method [23] with slight modifications. Two grams of crushed FD tofu was suspended in 38 mL of 0.2 M HCl containing 0.2% butylated hydroxytoluene (0.2 mL) and distilled until the total volume reached 50 mL. A 3 mL of the aliquot was mixed with 3 mL of 5 mM 2-thiobabituric acid (dissolved in 50% acetic acid; Sigma-Aldrich, St. Louis, MO, USA), reacted at 90 °C for 30 min, and the optical density was measured at 538 nm using a Thermo Multiskan Spectrum spectrophotometer (Thermo Scientific, Waltham, MA, USA). The TBA value was determined using a standard curve of 1.56–25 µg/mL malonaldehyde bis (MA; Sigma-Aldrich), and the value was calculated as milligrams of equivalent MA per kilogram of the sample.

### 2.11. Surface Color

The surface color of FD tofu was measured using a portable colorimeter (CR-300; Minolta Co., Ltd., Osaka, Japan). Data are presented as lightness (L*), redness (a*), and yellowness (b*) values according to Commission Internationale de l’Eclairage (CIE) color coordinates.

### 2.12. Data Analysis

All data were statistically assessed using the IBM SPSS Statistics program 20 (IBM, Armonk, NY, USA). All the experiments were conducted in triplicate. The values are expressed as the mean ± standard deviation (SD), and a significant difference (*p* < 0.05) in the means was identified using Tukey’s test.

## 3. Results and Discussion

### 3.1. Yields of Tofu and FD Tofu

**Figure 1:**
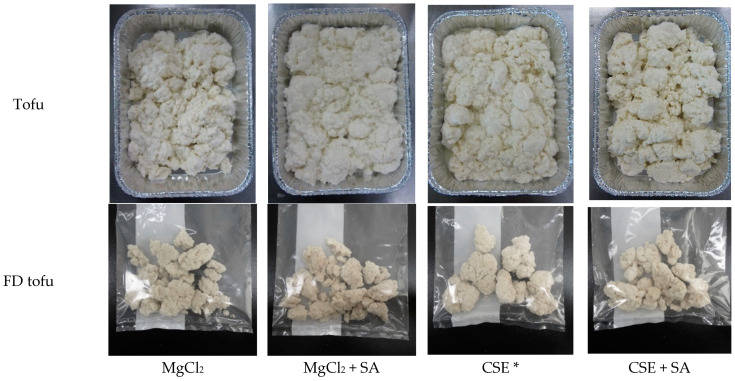
Appearances of tofu and FD tofu samples with MgCl_2_ or CSE according to SA addition. * Crab shell extract.

The quality and yield of tofu can be affected by various factors such as the soybean cultivar (protein content), processing conditions, and coagulant concentration [24]. In general, tofu preparation includes a molding process wherein tofu is pressed; thus, firmness increases with water release [25]. However, the tofu used in this study did not undergo the molding process; it is a traditional Korean tofu (Soondubu) that has a higher moisture content and softer texture than pressed tofu. The appearances of the tofu and FD tofu are shown in Figure 1. 

The yields of tofu and FD tofu samples with MgCl_2_ or CSE following the additions of SA are listed in Table 1. The yields of tofu samples were 246.7–256.3 g from 100 g of soybeans. After freeze-drying, the tofu samples weighed 48.3–49.9 g. There were no significant differences according to coagulant type or SA addition (*p* < 0.05).

### 3.2. Brokenness and Hardness of FD Tofu

**Figure 2:**
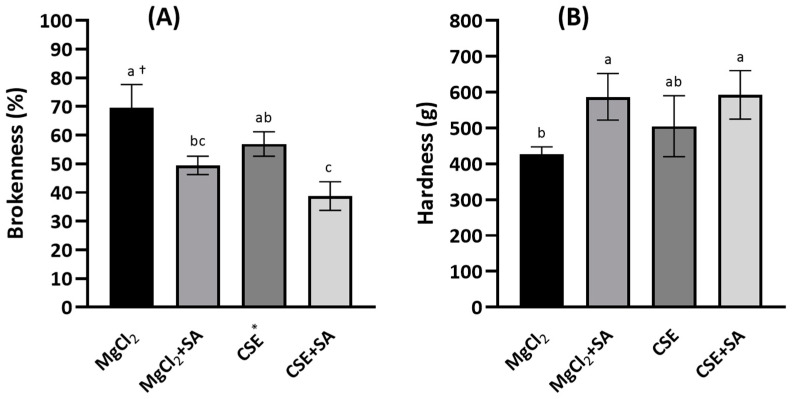
Brokenness (**A**) and hardness (**B**) of the FD tofu samples with MgCl_2_ or CSE according to SA addition. * Crab shell extract. Data expressed as the mean ± SD in triplicate. ^†^ The different letters indicate significantly different values within the experimental groups (*p* < 0.05).

Brokenness was determined by thumping with a stomacher because FD tofu was too light to be measured using a general friabilator apparatus. Brokenness was lower in FD tofu coagulated with CSE (57.0%) than in FD tofu coagulated with MgCl_2_ (69.6%); however, the difference was not significant (*p* < 0.05) (Figure 2A). Brokenness was significantly reduced in FD tofu samples coagulated with MgCl_2_ or CSE with the addition of SA (*p* < 0.05). In contrast, the hardness of FD tofu with CSE (592.4 g) was higher than that with MgCl_2_ (505.3 g), and the hardness of both tofu samples was notably increased by the addition of SA (Figure 2B).

Alginate is a colloidal polysaccharide that increases gel strength by cross-linking with divalent cations. This property might affect the gel-forming ability with the increasing hardness of tofu, although it depends on the composition and arrangement of α-L-guluronic acid and β-D-mannuronic acid, including their molecular size and distribution [7,26]. Tofu preparation involves protein denaturation by heat, wherein proteins are unfolded and form protein filaments owing to the formation of disulfide bonds, hydrophobic interactions, and hydrophobic coagulation promoted by a coagulant [27,28]. Hsiao et al. [6] studied the effect of propylene glycol alginate (PGA) on the coacervation of the conclycin (7S) and glycin (11S) proteins and isoflavones in heated soymilk for use as a potent coagulant. In their study, 0.9% PGA induced the coacervation of the 7S and 11S proteins and isoflavones. In contrast, carrageenan addition notably decreases the hardness of tofu coagulated with calcium sulfate or calcium acetate [29].

### 3.3. Water-Holding Capacities of Tofu and Rehydrated FD Tofu

**Figure 3:**
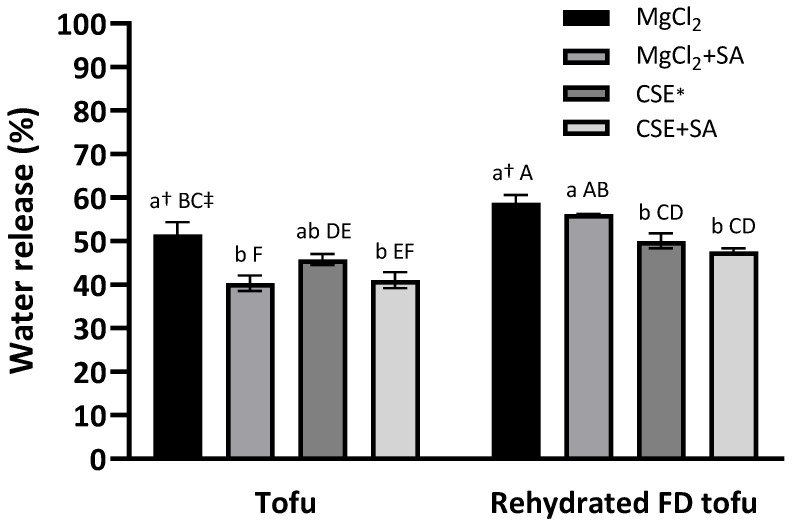
Water-holding capacities of the tofu and rehydrated FD tofu samples with MgCl_2_ or CSE according to SA addition. * Crab shell extract. Data expressed as the mean ± SD in triplicate. ^†^ The different lower case letters indicate significantly different values within tofu or rehydrated FD tofu (*p* < 0.05). ^‡^ The different capital letters indicate significantly different values within all the experimental groups (*p* < 0.05).

A low water release value indicates a high WHC. There was no significant difference in the tofu groups according to the coagulant type (*p* < 0.05), and water release in tofu and FD tofu samples was notably reduced by SA addition (Figure 3). This indicated that SA positively affected the WHC of tofu. The addition of alginate and calcium to the protein resulted in better water-holding capacity with a dual gel network and an interpenetrating network gel structure [13]. After freeze-drying, the water release increased and SA addition did not significantly reduce the water release (*p* < 0.05), whereas the water release exhibited a significant difference according to the coagulant type: the water release of the FD tofu samples with CSE (50.1%) or CSE + SA (47.6%) was lower than that of the FD tofu samples with MgCl_2_ (58.8%) or MgCl_2_ + SA (56.2%) (*p* < 0.05).

### 3.4. Textural Properties of Tofu and Rehydrated FD Tofu

Table 2 shows the textural properties of tofu and rehydrated FD tofu samples, which were characterized by hardness, springiness, cohesiveness, and gumminess. There was no statistical significance according to the coagulant type and SA addition in each comparison of the tofu group or rehydrated FD tofu group (*p* < 0.05). However, the hardness and gumminess of the rehydrated FD tofu group were higher than those of the non-FD tofu group. This might be related to the water absorption of tofu and matched well with the water release results. A decrease in the WHC of freeze-dried products after rehydration is often observed [30]. Water release was low in the rehydrated FD tofu samples treated with CSE or CSE + SA, and the differences in textural properties after freeze-drying were also lower than those of the rehydrated FD tofu samples treated with MgCl_2_ or MgCl_2_ + SA. This suggested that the CSE coagulant might increase the restorability of tofu after freeze-drying compared to MgCl_2_.

### 3.5. Microstructure of FD Tofu

**Figure 4:**
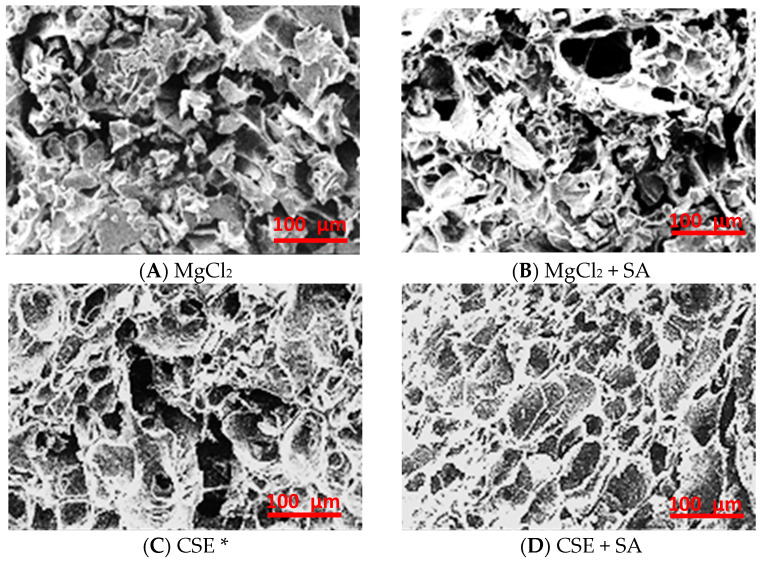
Microstructure of the FD tofu samples with MgCl_2_ (**A**), MgCl_2_ + SA (**B**), CSE (**C**), or CSE + SA (**D**) by scanning electron microscopy (SEM) at ×300 magnification. * Crab shell extract.

The shapes of the FD tofu microstructures were quite different depending on the coagulant; FD tofu with MgCl_2_ showed heterogeneous fractal aggregates, whereas FD tofu with CSE exhibited concatenate-layered aggregates that were like those of tofu coagulated with CaSO_4_ (Figure 4) [31]. The microstructure of FD tofu samples seemed to become protein-clumped aggregates with the addition of MgCl_2_ (Figure 4A) or CSE (Figure 4C), whereas both FD tofu and that with the addition of SA seemed to become a mesh structure with a few holes (Figure 4B,D). The denser network of FD tofu according to the addition of SA might support the water release results of rehydrated FD tofu samples in Figure 3. The protein and polysaccharide interaction could lead to a denser gel network, therefore, it is used as an enhancer for the structure, texture, and stability of food products [32]. Complexation of protein and polysaccharides induced by cross-linking between positively charged protein and negatively charged polysaccharides owing to the electrostatic interaction usually occurs below the isoelectric point of the proteins [33]. 

### 3.6. Change in the Brokenness of FD Tofu during Storage

**Figure 5:**
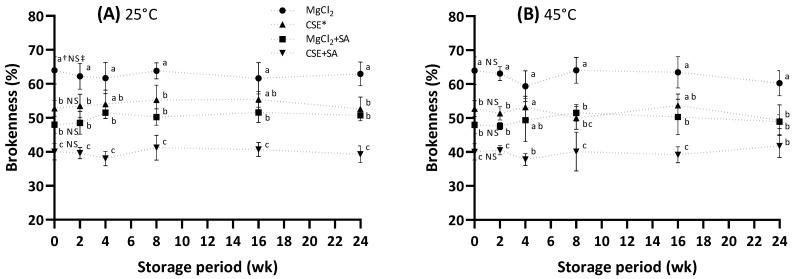
Change in the brokenness of the FD tofu samples according to the coagulant types and SA addition during storage at 25 °C (**A**) or 45 °C (**B**). * Crab shell extract. Data expressed as the mean ± SD in triplicate. ^†^ The different lower case letters indicate significantly different values between the experimental groups (*p* < 0.05). ^‡^ No significant difference within storage period (*p* < 0.05).

In general, FD food has a long storage stability at room temperature [34], but the FD tofu is only distributed within approximately 6 months without a cold chain. For this reason, the storage evaluation of the FD tofu was conducted at 25 °C and 45 °C for 6 months, and 45 °C is considered a high temperature by seasonal effects. The initial brokenness of the FD tofu sample (Figure 5) is accompanied by the results shown in Figure 2A. During storage, it was unchanged in all tofu samples, indicating that the tofu samples maintained their original structure during storage.

### 3.7. Changes in the VBN and TBA Values of FD Tofu during Storage

The FD tofu samples contained 4.0–6.5% ash, 54.6–56.6% protein, and 25.0–27.9% lipid, regardless of the coagulant type and SA addition. The VBN and TBA can be the main parameters to evaluate tofu shelf-life since tofu contains high protein and high lipid content [35]. Protein oxidation and lipid rancidity in freeze-dried foods during storage were also considered [36]. The average initial VBN value in all FD tofu samples was approximately 6.30 mg% (Table 3). The VBN values exhibited a different manner according to the temperature during storage; the VBN value of the FD tofu samples stored at 25 °C seemed to be retained at the initial value for 24 weeks, while a drastic increase was observed in all the FD tofu samples stored at 45 °C from 8 weeks. The TBA value gradually decreased during the storage period, regardless of the coagulant type or SA addition.

### 3.8. Changes in the Color Value of FD Tofu during Storage

The surface color of FD tofu was measured during storage and presented as lightness (L*), redness (a*), and yellowness (b*) values of the international color system (Table 4). Among the initial color values, there was only a significant difference in the a* value (*p* < 0.05), which was higher in FD tofu samples treated with CSE or CSE + SA than in FD tofu samples treated with MgCl_2_ or MgCl_2_ + SA. An increase in the L* value was observed in all tofu samples until 4 weeks, after which the decrease and increase tendency were repeated regardless of the coagulant type, SA addition, and storage temperature. 

Interestingly, a notable variation was observed in the b* value within the FD tofu samples stored at 45 °C; the values of the FD tofu samples with CSE or MgCl_2_ decreased, while the b* values for the FD tofu samples with SA addition were retained during the storage period. The a* values were retained for all the tofu samples stored at 25 °C, while those of all the tofu samples stored 45 °C tended to increase during storage. Low moisture content in freeze-dried food products can inhibit the growth of microorganisms and chemical reactions to some extent; however, it could lead to lipid oxidation, Maillard browning, and enzymatic reactions [37]. In general, the Maillard reaction occurs owing to many factors, including temperature, pH, water activity, and reactant types [38].

## 4. Conclusions

This study aimed to reduce FD tofu breakdown through improved physical properties with the addition of SA. To investigate the effects of sodium alginate on the physical properties of FD tofu with CSE, they were compared with FD tofu with MgCl_2_. The addition of SA notably reduced the brokenness of FD tofu and increased the hardness of both tofu samples by improving the network structure. Regarding the coagulant type, CSE was comparable to MgCl_2_ for all physical parameters. In particular, the water release of FD tofu samples with CSE (50.1%) or CSE + SA (47.6%) was lower than that of FD tofu samples with MgCl_2_ (58.8%) or MgCl_2_ + SA (56.2%) (*p* < 0.05). The brokenness of all tofu samples was unchanged for 6 months during storage at 25 °C or 45 °C. There were no significant differences in the VBN and TBA values according to the coagulant type and SA addition (*p* < 0.05). The FD tofu with CSE showed better physical properties than tofu with MgCl_2_, and the addition of SA reduced the brokenness of FD tofu, which can be a strategy for improving the quality of FD tofu and may decrease economic losses.

## Figures and Tables

**Table 1:** Yields of tofu and FD tofu with MgCl_2_ or CSE according to SA addition.

Group	Yield of Tofu (g 100 g^−1 †^)	Moisture Content (g 100 g^−1^)	pH of Soymilk	Yield of FD Tofu (g 100 g^−1 †^)
MgCl_2_	246.7 ± 4.6 ^ns‡^	79.5 ± 1.3 ^ns^	6.38 ± 0.06 ^ns^	49.9 ± 2.4 ^ns^
MgCl_2_ + SA	254.5 ± 7.1	80.1 ± 2.2	6.38 ± 0.06	49.9 ± 4.2
CSE *	256.4 ± 5.3	80.9 ± 0.7	6.31 ± 0.01	48.3 ± 0.9
CSE + SA	253.3 ± 5.7	80.4 ± 1.6	6.32 ± 0.04	48.9 ± 2.9

* Crab shell extract. ^†^ Soybean weight. The data are expressed as the mean ± SD in triplicate. ^‡^ No significant difference in the vertical column (*p* < 0.05). Tofu was prepared from 750 mL soymilk (125 g soybean) with 2.5 g coagulant and 0.375 g SA.

**Table 2:** Textural properties of tofu and rehydrated FD tofu samples with MgCl_2_ or CSE according to SA addition.

Group		Hardness (g)	Springiness	Cohesiveness	Gumminess
Tofu	MgCl_2_	23.6 ± 2.8 ^ns† CD‡^	1.0 ± 0.0 ^ns NS§^	0.6 ± 0.1 ^ns NS^	13.3 ± 4.7 ^ns C^
	MgCl_2_ + SA	23.4 ± 2.0 ^CD^	1.0 ± 0.1	0.7 ± 0.0	14.5 ± 1.4 ^BC^
	CSE *	20.0 ± 2.4 ^D^	1.0 ± 0.0	0.7 ± 0.0	19.2 ± 2.6 ^BC^
	CSE + SA	24.8 ± 1.3 ^CD^	1.0 ± 0.0	0.6 ± 0.1	19.5 ± 3.8 ^BC^
Rehydrated	MgCl_2_	47.3 ± 5.1 ^ns† AB^	1.0 ± 0.3 ^ns^	0.7 ± 0.1 ^ns^	34.9 ± 6.9 ^ns A^
FD tofu	MgCl_2_ + SA	49.4 ± 7.9 ^A^	1.0 ± 0.1	0.8 ± 0.1	36.9 ± 7.3 ^A^
	CSE	35.7 ± 4.7 ^BC^	1.0 ± 0.1	0.8 ± 0.1	26.9 ± 3.8 ^AB^
	CSE + SA	39.7 ± 6.1 ^AB^	1.1 ± 0.1	0.7 ± 0.1	27.2 ± 0.6 ^AB^

* Crab shell extract. The data are expressed as the mean ± SD in quintuplicate. ^†^ No significant difference within tofu or rehydrated FD tofu samples (*p* < 0.05). ^‡^ The different capital letters indicate significantly different values within all the experimental groups (*p* < 0.05). ^§^ No significant difference within all the experimental groups (*p* < 0.05).

**Table 3:** Changes in the volatile base nitrogen (VBN) (A) and thiobarbituric acid (TBA) (B) values of FD tofu samples according to the coagulant types and SA addition during storage at 25 °C or 45 °C.

	Temp.	Group	Day					
			0	2	4	8	16	24
VBN (mg%)	25 °C	MgCl_2_	6.28 ± 0.89 ^ns† NS‡^	6.96 ± 0.36 ^ns^	6.25 ± 0.98 ^ns^	7.68 ± 0.99 ^ns^	8.31 ± 1.96 ^ns^	9.46 ± 1.91 ^ns^
		MgCl_2_ + SA	6.30 ± 0.99 ^NS^	7.69 ± 2.37	7.64 ± 0.88	8.18 ± 0.24	8.38 ± 1.97	9.08 ± 1.02
		CSE *	6.32 ± 0.68 ^NS^	7.59 ± 0.98	7.64 ± 0.98	7.70 ± 1.14	8.27 ± 1.95	9.79 ± 3.96
		CSE + SA	6.29 ± 1.02 ^NS^	6.29 ± 1.39	6.94 ± 1.97	6.99 ± 0.42	7.70 ± 0.98	9.78 ± 1.68
	45 °C	MgCl_2_	6.39 ± 1.21 ^ns† C§^	6.30 ± 0.80 ^ns C^	9.03 ± 0.98 ^ns BC^	12.59 ± 1.98 ^ns AB^	11.76 ± 0.98 ^ns AB^	13.92 ± 1.97 ^ns A^
		MgCl_2_ + SA	6.33 ± 0.86 ^D^	7.00 ± 1.68 ^CD^	8.33 ± 1.26 ^BCD^	10.49 ± 0.99 ^ABC^	12.98 ± 0.97 ^A^	11.87 ± 1.39 ^AB^
		CSE	6.28 ± 1.38 ^C^	6.99 ± 0.36 ^BC^	8.38 ± 1.97 ^ABC^	10.55 ± 2.97 ^ABC^	11.15 ± 1.60 ^AB^	11.89 ± 1.00 ^A^
		CSE + SA	6.36 ± 0.98 ^B^	7.69 ± 0.99 ^B^	8.34 ± 1.87 ^B^	10.69 ± 1.21 ^AB^	13.18 ± 2.94 ^A^	12.93 ± 0.49 ^A^
TBA (mgMAeq kg^−1^)	25 °C	MgCl_2_	0.56 ± 0.01 ^a¶ A^	0.54 ± 0.02 ^ns† AB^	0.52 ± 0.02 ^ns BC^	0.54 ± 0.02 ^ns AB^	0.47 ± 0.01 ^ns CD^	0.45 ± 0.01 ^a D^
		MgCl_2_ + SA	0.53 ± 0.02 ^ab A^	0.49 ± 0.01 ^AB^	0.50 ± 0.03 ^AB^	0.54 ± 0.03 ^A^	0.45 ± 0.01 ^B^	0.45 ± 0.01 ^a B^
		CSE	0.52 ± 0.01 ^b A^	0.49 ± 0.03 ^AB^	0.50 ± 0.03 ^AB^	0.52 ± 0.03 ^AB^	0.45 ± 0.03 ^BC^	0.41 ± 0.02 ^b C^
		CSE + SA	0.53 ± 0.01 ^ab A^	0.49 ± 0.03 ^AB^	0.46 ± 0.03 ^ABC^	0.51 ± 0.03 ^AB^	0.45 ± 0.02 ^BC^	0.42 ± 0.02 ^ab C^
	45 °C	MgCl_2_	0.56 ± 0.01 ^a A^	0.56 ± 0.02 ^a A^	0.55 ± 0.02 ^a A^	0.53 ± 0.02 ^ab AB^	0.51 ± 0.01 ^ab B^	0.44 ± 0.01 ^ns† C^
		MgCl_2_ + SA	0.53 ± 0.02 ^ab A^	0.54 ± 0.01 ^ab A^	0.52 ± 0.01 ^ab A^	0.55 ± 0.02 ^a A^	0.52 ± 0.01 ^a A^	0.47 ± 0.01 ^B^
		CSE	0.52 ± 0.01 ^b A^	0.51 ± 0.03 ^ab A^	0.48 ± 0.03 ^b AB^	0.49 ± 0.02 ^b AB^	0.49 ± 0.01 ^b AB^	0.45 ± 0.02 ^B^
		CSE + SA	0.53 ± 0.01 ^ab A^	0.49 ± 0.02 ^b AB^	0.48 ± 0.03 ^b BC^	0.51 ± 0.01 ^ab AB^	0.51 ± 0.01 ^ab AB^	0.44 ± 0.02 ^B^

* Crab shell extract. Data expressed as the mean ± SD in triplicate. ^†^ No significant difference within 25 °C or 45 °C (*p* < 0.05). ^‡^ No significant difference within all the experimental groups (*p* < 0.05). ^§^ The different capital letters indicate significantly different values within storage period (*p* < 0.05). ^¶^ The different lower case letters indicate significantly different values within 25 °C or 45 °C (*p* < 0.05).

**Table 4:** Changes in the L*, a*, and b* values of the FD tofu samples according to the coagulant types and SA addition during storage at 25 °C or 45 °C.

	Temp.	Group	Day					
			0	2	4	8	16	24
L* value	25 °C	MgCl_2_	92.7 ± 0.2 ^ab† BC‡^	93.3 ± 0.6 ^ns§ AB^	93.8 ± 0.1 ^ns A^	93.1 ± 0.3 ^a AB^	93.1 ± 0.2 ^ns AB^	92.1 ± 0.3 ^b B^
		MgCl_2_ + SA	93.1 ± 0.2 ^a AB^	93.1 ± 0.1 ^AB^	93.6 ± 0.1 ^A^	92.7 ± 0.3 ^ab BC^	92.7 ± 0.2 ^BC^	92.2 ± 0.2 ^b C^
		CSE *	92.5 ± 0.3 ^b B^	92.9 ± 0.3 ^AB^	93.5 ± 0.3 ^A^	92.4 ± 0.3 ^b B^	92.9 ± 0.1 ^AB^	92.9 ± 0.1 ^a AB^
		CSE + SA	92.8 ± 0.1 ^ab B^	92.7 ± 0.3 ^B^	93.6 ± 0.2 ^A^	92.5 ± 0.1 ^ab BC^	92.8 ± 0.1 ^B^	92.3 ± 0.1 ^b C^
	45 °C	MgCl_2_	92.7 ± 0.2 ^ab B^	93.3 ± 0.1 ^a A^	93.7 ± 0.1 ^ns A^	92.8 ± 0.2 ^ns B^	92.3 ± 0.1 ^b C^	92.5 ± 0.1 ^ns BC^
		MgCl_2_ + SA	93.1 ± 0.2 ^a AB^	92.9 ± 0.1 ^b BC^	93.4 ± 0.1 ^A^	92.5 ± 0.2 ^CD^	92.8 ± 0.1 ^a BCD^	92.4 ± 0.1 ^D^
		CSE	92.5 ± 0.3 ^b B^	92.9 ± 0.1 ^b AB^	93.5 ± 0.1 ^A^	92.8 ± 0.1 ^B^	92.8 ± 0.3 ^a B^	92.7 ± 0.3 ^B^
		CSE + SA	92.8 ± 0.1 ^ab BC^	93.0 ± 0.1 ^b B^	93.5 ± 0.2 ^A^	92.6 ± 0.2 ^BC^	92.4 ± 0.1 ^ab C^	92.6 ± 0.3 ^BC^
a* value	25 °C	MgCl_2_	−1.2 ± 0.2 ^ns B^	−1.2 ± 0.0 ^b AB^	−1.1 ± 0.1 ^b AB^	−1.2 ± 0.1 ^b AB^	−1.1 ± 0.0 ^b AB^	−1.0 ± 0.0 ^ns A^
		MgCl_2_ + SA	−1.2 ± 0.0 ^B^	−1.2 ± 0.1 ^b B^	−1.2 ± 0.0 ^b B^	−1.2 ± 0.0 ^b B^	−1.1 ± 0.1 ^b B^	−1.0 ± 0.1 ^A^
		CSE	−1.0 ± 0.0 ^NS¶^	−1.0 ± 0.1 ^a^	−1.0 ± 0.1 ^a^	−1.0 ± 0.0 ^a^	−1.0 ± 0.1 ^a^	−1.0 ± 0.0
		CSE + SA	−1.0 ± 0.0 ^BC^	−1.0 ± 0.0 ^a AB^	−1.1 ± 0.0 ^ab BC^	−1.1 ± 0.0 ^ab C^	−1.0 ± 0.0 ^ab BC^	−0.9 ± 0.0 ^A^
	45 °C	MgCl_2_	−1.2 ± 0.2 ^ns C^	−1.1 ± 0.0 ^b BC^	−1.1 ± 0.0 ^c BC^	−1.0 ± 0.0 ^ns BC^	−0.7 ± 0.2 ^ns A^	−0.8 ± 0.0 ^b AB^
		MgCl_2_ + SA	−1.2 ± 0.0 ^D^	−1.1 ± 0.0 ^b C^	−1.0 ± 0.0 ^bc BC^	−0.9 ± 0.0 ^B^	−0.7 ± 0.0 ^A^	−0.8 ± 0.0 ^b A^
		CSE	−1.0 ± 0.0 ^C^	−1.0 ± 0.0 ^a C^	−0.9 ± 0.0 ^a B^	−1.0 ± 0.0 ^C^	−0.6 ± 0.1 ^A^	−0.6 ± 0.0 ^a A^
		CSE + SA	−1.0 ± 0.0 ^B^	−1.0 ± 0.0 ^ab B^	−1.0 ± 0.0 ^b B^	−1.0 ± 0.0 ^B^	−0.7 ± 0.0 ^A^	−0.6 ± 0.0 ^a A^
b* value	25 °C	MgCl_2_	13.0 ± 0.3 ^ns NS^	13.0 ± 0.4 ^ns^	12.9 ± 0.0 ^ns^	13.1 ± 0.5 ^ns^	13.1 ± 0.3 ^ns^	13.0 ± 0.3 ^ns^
		MgCl_2_ + SA	13.5 ± 0.2 ^NS^	13.6 ± 0.2	13.1 ± 0.1	13.3 ± 0.3	13.5 ± 0.5	13.3 ± 0.3
		CSE	13.4 ± 0.3 ^NS^	13.3 ± 0.4	13.1 ± 0.2	13.4 ± 0.1	13.5 ± 0.1	13.0 ± 0.2
		CSE + SA	13.7 ± 0.0 ^A^	13.7 ± 0.3 ^A^	13.1 ± 0.3 ^AB^	13.6 ± 0.5 ^AB^	13.6 ± 0.1 ^A^	12.8 ± 0.0 ^B^
	45 °C	MgCl_2_	13.0 ± 0.3 ^ns A^	12.9 ± 0.5 ^b AB^	12.7 ± 0.2 ^ns AB^	12.4 ± 0.1 ^ns AB^	12.1 ± 0.4 ^b B^	12.0 ± 0.2 ^ns B^
		MgCl_2_ + SA	13.5 ± 0.2 ^AB^	13.7 ± 0.1 ^a A^	12.9 ± 0.1 ^BC^	12.8 ± 0.2 ^BC^	12.8 ± 0.1 ^ab BC^	12.7 ± 0.5 ^C^
		CSE	13.4 ± 0.3 ^A^	13.4 ± 0.1 ^ab A^	12.4 ± 0.2 ^B^	12.4 ± 0.5 ^B^	12.3 ± 0.4 ^ab B^	12.4 ± 0.3 ^B^
		CSE + SA	13.7 ± 0.0 ^AB^	13.9 ± 0.3 ^a A^	12.8 ± 0.3 ^C^	12.7 ± 0.3 ^C^	13.0 ± 0.2 ^a BC^	12.8 ± 0.4 ^C^

* Crab shell extract. Data expressed as the mean ± SD in triplicate. ^†^ The different lower case letters indicate significantly different values within 25 °C or 45 °C (*p* < 0.05). ^‡^ The different capital letters indicate significantly different values within storage period (*p* < 0.05). ^§^ No significant difference within 25 °C or 45 °C (*p* < 0.05). ^¶^ No significant difference within storage period (*p* < 0.05).

## Data Availability

Data presented in this study are available in the article.
